# Near-field surface plasmon field enhancement induced by rippled surfaces

**DOI:** 10.3762/bjnano.8.97

**Published:** 2017-04-28

**Authors:** Mario D’Acunto, Francesco Fuso, Ruggero Micheletto, Makoto Naruse, Francesco Tantussi, Maria Allegrini

**Affiliations:** 1 CNR-ISM, Istituto di Struttura della Materia, Consiglio Nazionale delle Ricerche, via Fosso del Cavaliere 100, 00133, Rome, Italy; 2 CNR-IBF, Istituto di Biofisica, Consiglio Nazionale delle Ricerche, via Moruzzi 1, 56124, Pisa, Italy; 3 Dipartimento di Fisica Enrico Fermi and CNISM, Università di Pisa, Largo Bruno Pontecorvo 3, 56127, Pisa, Italy; 4 CNR-INO, Istituto Nazionale di Ottica, via Moruzzi 1, 56124, Pisa, Italy; 5 Yokohama City University, Graduate School of Nanobioscience, 22-2 Seto, Kanazawa-ku, Yokohama, Japan; 6 Harvard Medical School, 64 Sydney Street, Suite 170, Cambridge, 02139 MA, USA,; 7 Network System Research Institute, National Institute of Information and Communications Technology, 4-2-1 Nukui-kita, Koganei, Tokyo 184-8795, Japan

**Keywords:** aperture scanning near-field optical microscopy, gold rippled surface, localized hot spots, metal–dielectric−metal nanogaps, surface plasmon resonance

## Abstract

The occurrence of plasmon resonances on metallic nanometer-scale structures is an intrinsically nanoscale phenomenon, given that the two resonance conditions (i.e., negative dielectric permittivity and large free-space wavelength in comparison with system dimensions) are realized at the same time on the nanoscale. Resonances on surface metallic nanostructures are often experimentally found by probing the structures under investigation with radiation of various frequencies following a trial-and-error method. A general technique for the tuning of these resonances is highly desirable. In this paper we address the issue of the role of local surface patterns in the tuning of these resonances as a function of wavelength and electric field polarization. The effect of nanoscale roughness on the surface plasmon polaritons of randomly patterned gold films is numerically investigated. The field enhancement and relation to specific roughness patterns is analyzed, producing many different realizations of rippled surfaces. We demonstrate that irregular patterns act as metal–dielectric–metal local nanogaps (cavities) for the resonant plasmonic field. In turn, the numerical results are compared to experimental data obtained via aperture scanning near-field optical microscopy.

## Introduction

Metal nanostructures capable of producing localized surface plasmon polaritons (SPPs) are of fundamental relevance to study near-field nonlinear optical phenomena [1–2]. Particularly relevant is the strong electric field enhancement on resonance that can be of special interest for various applications, such as surface-enhanced Raman spectroscopy (SERS) [3–4], tip-enhanced Raman spectroscopy (TERS) [5], plasmonic photovoltaics [6–8], plasmonic nanosensors [9–10], and near-field optical theory [2,11–12]. It is commonly accepted that enormous field enhancements at the resonance of the optical response applied to randomly patterned metal nanostructures are highly dependent upon the optical excitation of SPPs, which are ultimately collective optical electromagnetic modes strongly connected to the nanostructure geometry and size [12]. This is because at a metal–dielectric interface, large electric field fluctuations can occur for a plasmon resonance frequency, ω_r_, that in a two-dimensional system corresponds to the condition Re(ε_m_(ω_r_)) = −ε_d_, where ε_m_(ω_r_) is the dielectric function of the metal at the resonant frequency and ε_d_ is the effective dielectric constant. In a randomly organized nanostructure, collective plasmon oscillations are deeply influenced by the locally irregular geometry, generating hot and cold spots corresponding to areas of high and low local fields, respectively. Local enhancements in the hot spot regions overcome the average surface enhancement by many orders of magnitude (relative magnitudes up to 10^5^ for the linear regime and up to 10^20^ for the nonlinear response have been reported [12]). Hot spots can be detected because the local enhancement peaks are generally spatially separated by distances much larger than the typical distance between neighboring hot spots [12].

The parameter characterizing the enhancement of local field intensity along one specific direction (*z* in our case, i.e., the orthogonal direction with respect to the substrate) is defined as [12]

[1]
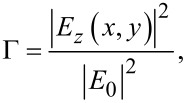


where *E*_0_ is the incident electric field. Owing to its nonlinear character, the enhancement of Raman scattering is quantified by taking the fourth power of the expression in Equation 1. Here, we obtain and plot the local enhancement parameter, Γ, for a wide variety of patterned surfaces. In particular, we will focus on a specific class of gold nanostructures featuring a rippled surface (which have already demonstrated their potential as SERS substrates) as well as their nonlinear optical properties [13–14].

Although the occurrence of surface plasmon-enhanced nonlinear optical effects is rather well understood, further investigation into the spectral dependence and magnitude dependence of the field enhancement as related to surface morphology [2]. Particular attention has been paid to the electric field distribution, because distribution and intensity of the hot spots have a tunable coherence length [13–15]. Not only the patterns, but also the spatial locations of the electric field peaks strongly depend on the frequency, whereby the Γ parameter typically decreases with the applied frequency. By changing the frequency, one can excite different nanometer-sized hot spots on a film. This effect has high potential for various applications in SERS, plasmon-enhanced photocatalysis and sensing applications [6–15]. In addition, polarization plays a fundamental role, in particular whenever the nanostructure morphology shows anisotropy at the local scale. Polarization can induce strong confinement of plasmons in the resonance region in close correlation with the local surface morphology, characterized by a pattern of hills and valleys. As a consequence, an accurate knowledge of the spatial distribution of large field enhancements requires an accurate knowledge of the nanostructure morphology. This is particularly true for the spatial distribution of a random pattern composed of a texture of hills and valleys. Rippled surfaces represent a special case of rough surfaces. Ripple formation due to light–matter interaction, for example, using short laser pulses, is highly dependent on the laser irradiation conditions (angle of incidence, polarization, laser power, scanning speed – only to cite the most important factors), material properties, environmental conditions, temperature, etc. It is commonly accepted that ripples formed as a consequence of light–matter interaction present a periodicity that is dependent on the polarization of the incident light. In addition, the formation of ripples by ultrashort laser pulses (<1 ps) enables fabrication on solid materials ranging from metals to transparent glasses and crystals [16]. As a consequence, the interest in the optical properties of rippled surfaces has a long history [17]. On the nanoscale, nanofabrication techniques, such as conventional lithographic methods or scanning probe techniques, allow for the production of predesigned rough surfaces, where simple and prescribed shapes can be easily produces such as rectangular or sinusoidal shapes, for example. Random surfaces, on the contrary, are stochastic and are a result of a (or several) random process(es). Nanofabrication techniques based on growth processes or self-organization have been demonstrated to be an excellent and relatively low-cost alternative, allowing maskless patterning of macroscopic surface areas [18]. Many of such techniques lead to typical patterns including fractal surfaces. Regular, or nearly regular, nanoscale ripples have width and height much smaller than the wavelength of typical plasmon resonances.

Different top-down or bottom-up fabrication techniques have been introduced to produce metal nanostructures with active plasmonic reactivity [14]. For example, ion beam sputtering (IBS) is a widely employed bottom-up technique that is a quick and low cost method for the synthesis of both large area arrays of self-organized nanowires for SERS molecular detection [14,19–20] and plasmon-enhanced photon harvesting in the vis–NIR range [14,21]. Other techniques widely employed to produce patterned metal nanostructures are top-down lithographic techniques such as electron beam lithography and nanosphere lithography [22–24].

In this paper, we simulate the conditions of SPP field enhancement and the formation of dark and hot, or bright spots, for a wide variety of patterned surfaces. The patterned surfaces are numerically generated, and the effects of roughness on the near-field scattered electric fields for different wavelengths falling in the optical range and for *s*- or *p*-polarization are evidenced using a system of integral equations, resulting from the application of Green’s theorem to the electromagnetic field equations.

## Modeling

### Influence of surface roughness on interference patterns of surface plasmons: near-field optical properties

The need to include the surface roughness into numerical models for plasmonic systems restricts the choice of numerical near-field methods [25–28]. Commonly recognized methods to describe the roughness of scattering objects include the finite element method (FEM) [29], the finite difference time domain (FDTD) method [30], the coupled wave method (CWM) [31], the discrete dipole approximation (DDA) [32–33], for which the meshing of the rough surface may be critical for computation. Our approach follows the so-called surface integral equations (SIEs) [34–35] approach. Initially, we implemented the SIEs based on the boundary element method (BEM), where ad-hoc integral equations for the surface charge and current density must be considered [36]. A second approach was based on a formulation of SIEs, resulting from application of Green’s theorem on the scattering volumes directly to the exact electromagnetic equations [37]. The latter is the approach followed to derive the numerical results presented in this paper [38]. The basic advantage of an approach based on the dyadic Green’s function is that the surface under investigation can be discretized into small volume elements, and hence, the optical response of two different volumes (also for complex morphologies) is mediated by the dyadic Green’s function [39].

**Figure 1:**
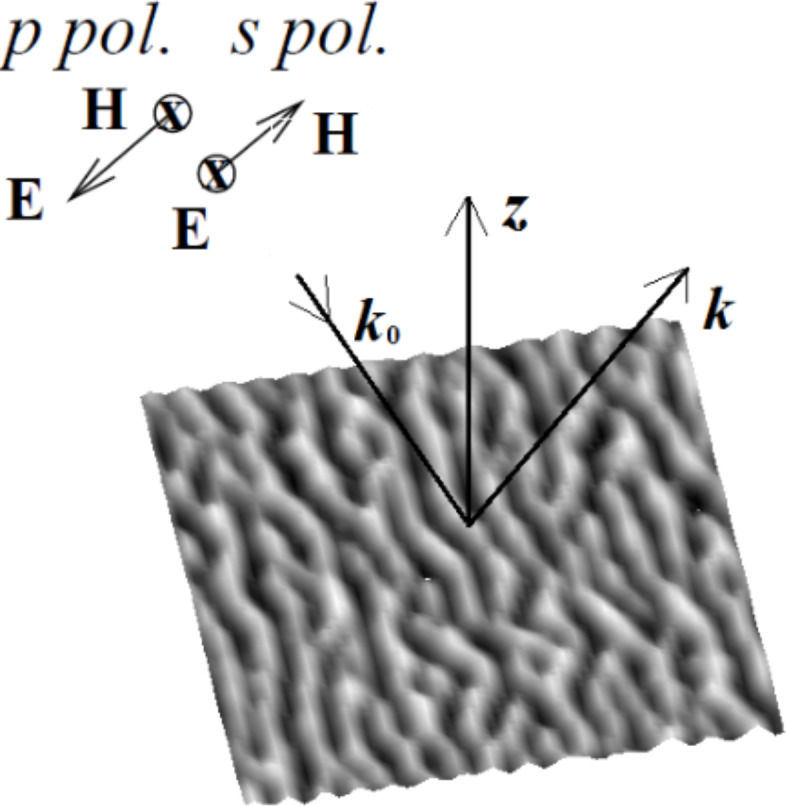
Schematic of the scattering geometry with the electromagnetic field vectors for linear *p*- and *s*- polarization. The image represents the typical morphology of the substrates in our calculations.

Near-field optical properties of rough metallic surfaces can be studied by illuminating the sample with an external light source at frequency ω. The scattering geometry is illustrated in Figure 1A where a rough metal surface with *z* = *h*(*x*,*y*) denotes the substrate, and where the semi-infinite metal occupies the lower half-space *z* ≤ *h*(*x*,*y*) which is characterized by an isotropic, homogeneous, frequency-dependent dielectric function ε^<^(ω). The medium of incidence, *z* > *h*(*x*,*y*), is characterized by a frequency-dependent dielectric function ε^>^(ω), and a monochromatic, linearly polarized, incident beam of frequency ω is assumed to impinge onto the interface at an angle θ, measured counterclockwise with respect to the positive *z* axis.

We now focus on the electric field calculation for the case of *p*-polarization. In order to obtain the electric field components, we can start with the Maxwell’s equation:

[2]
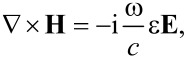


where the magnetic field in our system has the form:

[3]
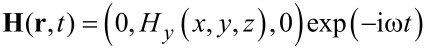


and the electric field is

[4]
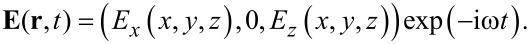


Given 
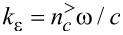
, 
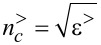
 and


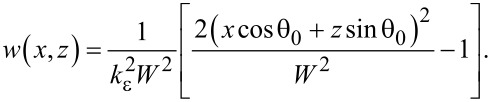


a monochromatic incident field (Gaussian beam with full-width half-max of *W*) of frequency ω can be expressed as in Equation 5 (considering, for the sake of simplicity, a *p*-polarized field) [40].

[5]
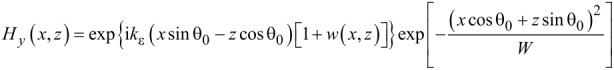


Since a time harmonic dependence e^−iω^*^t^* is assumed, the functional dependence on frequency will be omitted. Using Equation 2, and inserting the magnetic field component from Equation 3, we can write the electric field components as shown in Equation 6.

[6]
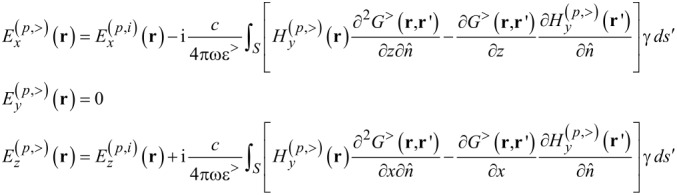


In Equation 6, the derivation of the Green’s function and the external potential, respectively, are made with respect to the unit vector along *z*.

[7]
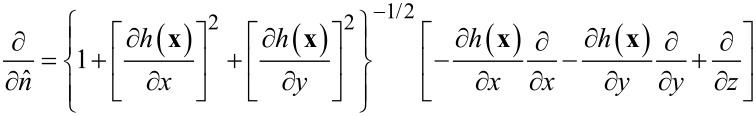


In Cartesian coordinates Equation 7 is the normal derivative to the rough surface with *z* = *h*(***x***), defined by the relation 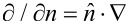, where 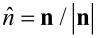 is a unit vector for


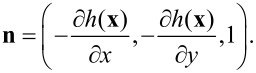


In the near-field, when **r** is very close to the surface, nonintegrable singularities can appear associated with Green’s functions derivatives. A way to work around this problem has been proposed by Sanchez-Gil et al. [40], where the evaluation of the electric field at the surface itself is made. In this case a simple relation connecting the normal and tangential components of the electric field with the surface magnetic field and its normal derivative can be found. The surface electric fields are obtained from the surface magnetic fields as follows [35,40]:

[8]
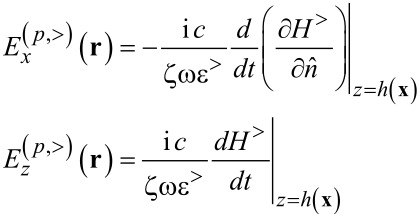


where


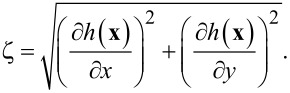


The calculation of the derivative Green’s function in Equation 6 requires an adequate strategy. The rough surface Green’s function is given by the sum of the coherent and incoherent contributions, where *G*_0_ is the coherent contribution and *G*_f_ is addressed by the random roughness. The coherent contribution can be expressed by [41]:

[9]
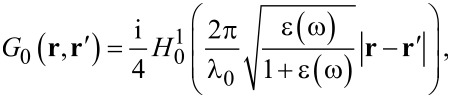


where 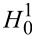 is the Hankel function of first kind and order zero, and ε(ω) is the metal electric permittivity (in our case, gold has been considered) described by the Drude model,

[10]
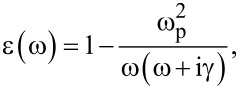


where ω_p_ = 2.183 × 10^15^ s^−1^ is the free-electron plasma frequency and γ *=* 1.41 × 10^14^ rad·s^−1^ is the relaxation rate. A possible approach to calculate the incoherent contribution is the so-called small-roughness approach, previously developed by Ishimaru et al. [42]. Following this approach, we develop a smoothing first-order approximation for the small-roughness regime. In the small-roughness regime, the Green’s function is given by the sum of the coherent contribution, *G*_0_, and the incoherent, fluctuating term, *G*_f_ [42]

[11]
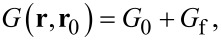


where the fluctuating term *G*_f_, can be developed by expanding the Green’s function around the surface height *h*(*x*). In Supporting Information File 1, we give a derivation of the fluctuating term for the Green function (Equation 11). Here, we summarize only the result

[12]
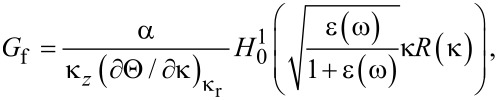


where κ_r_ denotes a spatial wavevector, and in particular, is the root of Θ(κ_r_) + 1 = 0 (Θ is a function linking the reflection coefficient to the spectral density and plasmon wavevector, see Supporting Information File 1), and α a numerical factor. The complete derivation of Equation 12 is detailed in Supporting Information File 1. It is evident that the singularities present in G_f_ and corresponding to the eigenmodes drive the plasmon resonance and the field enhancements. In fact, the pole accounts for all of the contribution to Green’s functions from the rough surface*.* Only under small-roughness conditions *G*_0_ ≈ *G*_f_, while in general *G*_f_ >> *G*_0_.

Now, we must define the ripple surfaces providing us the profile *h*(***x***) that we will use in the SIE picture. Here, we are using the notation that 
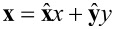
, where 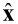 and 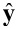 are unit vectors along the *x* and *y* directions. Since many patterning techniques, including ballistic deposition processes (such as molecular beam epitaxy or IBS) or plasma etching, are characterized by dynamic roughening that constitutes an important class of nonequilibrium phenomena [20,43]. In such systems, the dynamic roughening is generated by the fluctuation of incoming particles, and the rough surface progressively involves a columnar instability growth which generates typical patterns of grooves and hills, or transversal instability, which leads to the generation of longitudinal ripples or, again, longitudinal instability which leads to transversal ripples.

In the last decades several models have been proposed for describing the dynamic behavior of growing surfaces and corresponding patterns. In all such methods, the common approach is to asymptotically reduce the partial differential equations governing the complex growing dynamics to a sample equation for the interface [44–45]. One critical point in all such methods is represented by the choice of parameters to be included in the partial differential equations, and in many cases, such parameters must be chosen empirically. A unique possibility to simulate a wide range of rippled surfaces as generated by dynamic roughening is given by the Kuramoto–Sivashinsky (KS) equation [46]. This equation describes a near planar surface which is marginally long-wave unstable. We will use the KS equation to simulate the rippled surface with appropriate parameters to obtain the desired pattern. All details on the rippled surface simulated with a KS equation will be described in the next section. The simulated surface can be characterized by a variety of parameters, including, for instance, root mean square (rms), amplitude distribution function (i.e., the function that gives the probability that a roughness profile has a certain height at a position), autovariance and autocorrelation functions. The covariance measures how well two or more heights match one another. Simple analytical expressions for the height–height correlation can be obtained using a Gaussian function. The correlation gives a measure of the in-plane correlation length between the features detected directly in the topography surfaces. The surface profile function *h*(***x***) is a growing stochastic process described by the statistical properties

[13]
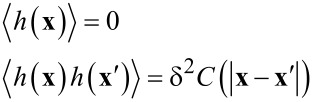


where the angular brackets denote an average over the ensemble of realizations of the surface profile, and δ^2^ is the mean-square offset of the surface from perfect flatness, δ^2^ = <*h*^2^(***x***)>, δ^2^ = 0 for a flat surface. Because the surfaces deposited by ion-beam sputtering (IBS) generally show a fractal structure, the function *C* in Equation 13 is chosen to be Gaussian, that is, a special case of a fractal surface with the Hurst exponent equal to one [47]:

[14]
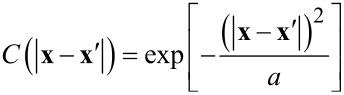


where *a* is known as the transverse autocorrelation length, as it describes the mean length between two hills or two valleys on the surface. Since the profile *h*(***x***) is a Gaussian-distributed random variable: (a) the average of the product of an odd number of factors of the profile with the same arguments vanishes, and (b) the average of the product of an even number of factors of the profile is given by the sum of the products of the averages of *h*(***x***)’s paired two-by-two in all possible ways. In turn, the effects of roughness can be explored taking into account wavevectors, which is made introducing the Fourier transform (FT) of the surface profile function. The FT of the autocovariance function gives the power spectral density function that shows the strength of the height variations as a function of frequency.

## Results and Discussion

The local field enhancement as given by Equation 1 is calculated by integrating Equation 6 on simulated rippled surfaces, where a suitable Green’s function (Equation 11) has been previously evaluated.

Essentially, three factors play a fundamental role in the enhancement of the electric field: the geometry, the applied wavelength and the polarization. The geometry defines the shape and the distribution of the irregular patterns which act as metal–dielectric–metal local nanogaps for the resonant plasmonic fields. In turn, the rippled shape of the surface corrugation makes the optical properties highly dependent on the polarization which can be switched between two directions, thus *p*- or *s*-polarizations have been considered.

Rippled surfaces describing growth processes subsequent to deposition rates as, for example, with the IBS technique or other equivalent patterning methods is a nonequilibrium process where a two-dimensional surface is defined by the height function *h*(*x*,*y*,*t*), whose evolution in time is monitored. The physics of the growth and of the subsequent patterning process can be described by a damped Kuramoto–Sivashinsky (DKS) equation [45–47]. We propose a finite-difference semi-implicit splitting scheme of second order in time and space to numerically solve the DKS equations, with phenomenological parameters. A Matlab code to solve the DKS and corresponding field enhancement has been written ad hoc. The stability of the numerical scheme is verified with time step and grid spacing tests for the pattern evolution. Surface realizations of length *L* = 2–3 μm consisting of *N* = 2048 sampling points were generated using a DKS equation with empirical parameters [43–44]. The resolution (*N* = 2048) is such that the minimum size is nearly 1 nm and the groove–groove distance (an equivalent of periodicity for more regular surfaces) is of approximately 100–200 nm (the autocorrelation length *a* in Equation 9) and height of 10–30 nm. The Au skin depth *d* = (*c*/ω)(−ε_m_)^−1/2^ in the optical range varies between approximately 20–50 nm in the vis-NIR range, so that the rippled surface can be considered a homogenous film of corrugated gold presenting a metal–dielectric interface with the air. The intercept of a Gaussian incident beam with the plane of the mean surface is kept constant regardless of the angle of incidence (*W*/cosθ_0_ = *L*/4, and θ_0_ = 45°) [38]. This intercept illuminates a sufficiently large region of the surface in terms of the incident wavelength range, nominally varying from 400 to 900 nm. The surface texture of the rippled surfaces considered here is composed of the interplay of patterns made of grooves, hills and valleys showing some degree of anisotropy. Alignment effects induced, for example, by the anisotropic ion bombardment can in fact take place, leading to the occurrence of one preferential direction. The surface plasmon polaritons are strongly dependent on such patterns and their characteristic lengths. In addition, linear polarization plays an important role because the irregularity of the grooves makes the polarization effectively only linear on the local scale.

**Figure 2:**
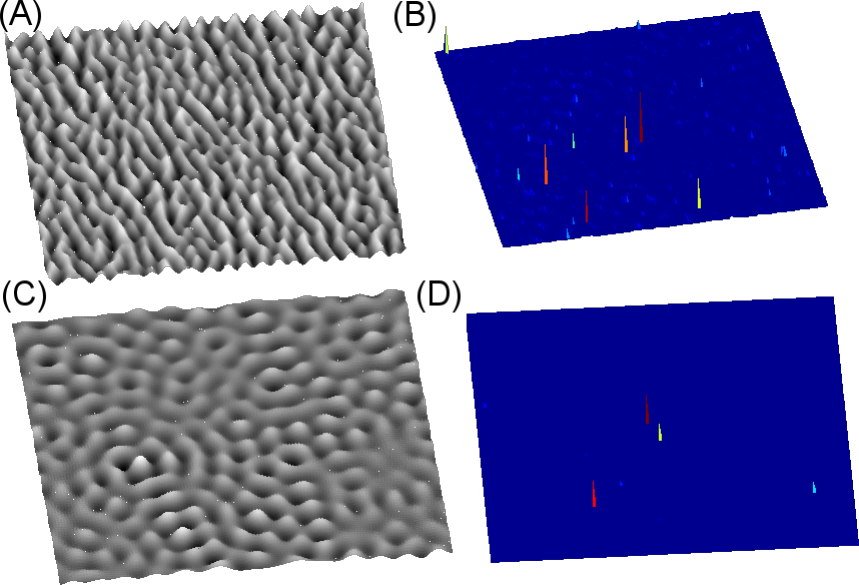
Results of the simulations described in the text. (A) We consider an intergroove distance (equivalent of the periodicity for more regular surfaces) with an average value around 150 nm and a height of 15−20 nm and (B) is its corresponding optical map with a maximum Γ factor of ≈10^7^. (C) Same conditions of (A) with the height set to approximately 10−20 nm and (D) is its corresponding optical map with a maximum Γ factor of ≈10^3^.

Once the ripple geometry can be reproduced, we can map the field enhancement factor that represents the hot spots eventually occurring for a specific illumination condition. In Figure 2, we consider two different rippled morphologies, (A) and (C), with corresponding SPP optical maps with enhancement factor Γ, shown in (B) and (D) respectively, for an applied wavelength λ = 785 nm under *p*-polarization conditions. Figure 2B and Figure 2D ultimately represent the distribution of the hot spots. Both reconstructed surfaces have a 2.5 × 2.5 μm area. Due to the highly localized nature of the hot spots, this area is representative of their spatial distribution. In the case of Figure 2A, we consider an intergroove distance (equivalent of the periodicity for more regular surfaces) with an average value around 150 nm and a height of 15–20 nm. In the case of Figure 2C, the height is approximately 10−20 nm. The Γ factor is peaked around ≈10^7^ and ≈10^3^ for the surface of Figure 2B and Figure 2D, respectively.

In particular, Figure 2A,C shows that rippled surfaces featuring a quasi-regular pattern of stripes, or nanowires, present a strong enhancement factor: the occurrence of two main peaks in Figure 2A makes impossible to detect (in the dynamics chosen for the representation) the weaker peaks. On the contrary, the holed rippled surface (Figure 2C) leads to a smaller enhancement factor by six orders of magnitude. Different from spherical geometries or regular nanowires, rippled surfaces, as in Figure 2, give the possibility to study the enhancement factor on surfaces presenting a wide range of nanogaps. It is well known that the gap between grooves plays a fundamental role in controlling the enhancement factor [12,48–49]. As a consequence, the random distribution of nanogaps in terms of shape and depth gives a unique possibility to study the distribution of field enhancement.

**Figure 3:**
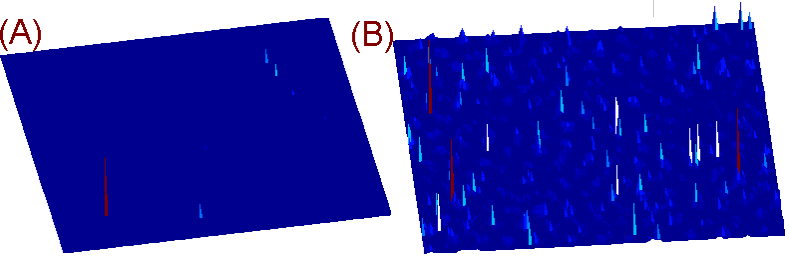
Maps of enhancement factor Γ for a realization of a rippled surface for (A) a wavelength of λ = 480 nm, with a maximum Γ ≈ 10^5^, and (B) λ = 785 nm, with a maximum Γ ≈ 10^11^.

To study the effects of local nanogaps, we first consider the role of the wavelength of the illumination field. In Figure 3, it is shown a comparison between the enhancement factor found for a rippled surface upon illumination at 480 and 785 nm (Figure 3A and Figure 3B, respectively). We are solely interested in demonstrating the difference and contrast for different frequencies, so we have chosen two convenient emission lines of our laser.

The correspondence between patterns of nanogaps, localized hot spots and polarization can be described by invoking the idea that the nanogaps are nanocavities able to trap the light at the cavity mode frequencies, and the physics of such light–matter interactions can be conveniently described in terms of the resonant cavity quasinormal modes (QNMs) [50]. QNMs are solutions of Maxwell’s equations that are associated with a complex eigenfrequency 
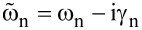
, where the imagery part is a measure of the energy leakage in the system, quantified by the quality factor *Q*_n_ = ω_n_/2γ_n_. To measure the spatial extent of localized QNMs, i.e., the surface occupied by the hot spot modes and their localization transition, we use the so-called inverse participation ratio, χ, that is also used in the theory of Anderson localization, defined as [51]

[15]
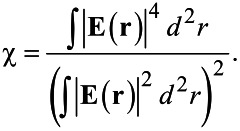


For spatially localized modes, χ is independent on the sample size *L*, whereas for extended modes, χ scales as *L*^−2^ [51]. In addition, for localized states, at a given point **r** and a given frequency ω, the electric field is dominated essentially by one mode*,* and the probability to find more than one mode given a high electric field is very small [52]*.* The inverse participation ratio is χ *≈* (*N*ξ*^2^*)*^−^*^1^, where *N* is the number of hot spots involved in the localized modes and ξ their characteristic localized length. A localized mode is characterized by *N* ≈ 1 hot spots with localized length ξ_l_ whereas delocalized modes involve *N* >> 1 hot spots, with average length ξ >> ξ_l_ so that for localized modes χ ≈ ξ_l_^−2^. Carminati et al. have shown that high values of the χ parameter correspond to large fluctuations of the local density of states (LDOSs) [52]. Each QNM is described by the Green’s functions, Equation 9 and Equation 12, that are closely related to the LDOSs. The LDOSs represents the weight of all normalized QNMs at a certain point of space for a certain light frequency and can be calculated by Equation 16.

[16]
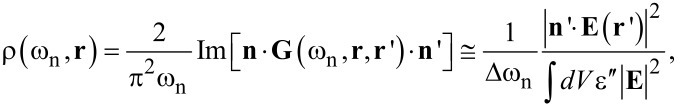


In Equation 16 **G** is the Green tensor, Δω_n_ is the spectral width of the mode and |**n·E**(**r**)|^2^ = *I*(**r**) its local intensity. Equation 16 summarizes the physical properties of nanogaps, showing that the electromagnetic field is enhanced when confined to a small volume. Hence, nanogaps can be considered as metal–insulator–metal (MIM) cavities characterized by the cavity *Q*-factor and the effective mode volume *V*_eff_, so that a large *Q*/*V*_eff_ ratio results in enhanced light–matter interaction, as typically quantified by the LDOS.

**Figure 4:**
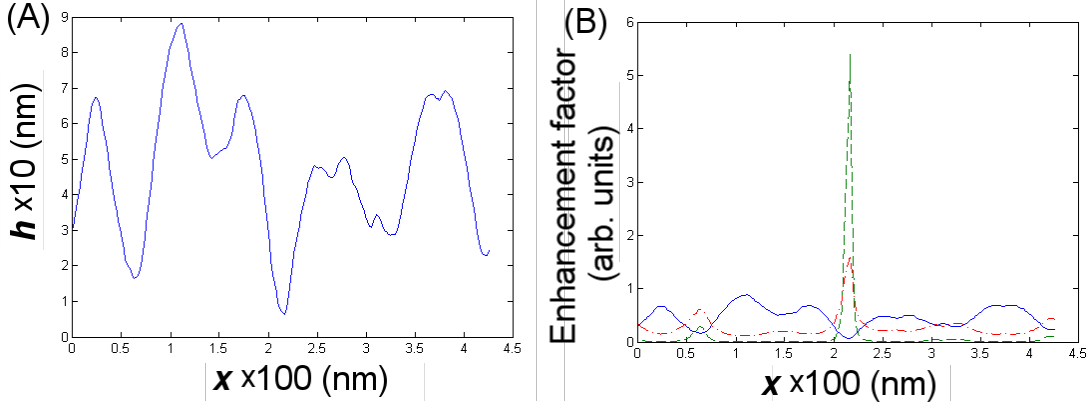
(A) The profile is extracted from the Figure 1A and represents a type of an aligned array of nanogaps. (B) The effect of polarization where the dashed curve (green) represents the field enhancement for an across-the-gap transverse polarization, with enhancement factor peak at ≈10^13^, and the dashed-dot curve (red) denotes the longitudinal polarization, enhancement peak ≈10^2^. The field enhancement on the *y*-scale is not to scale.

Let us consider a one-dimensional array of coupled QNMs, where the QNMs are exponentially localized with localization centers on the nanogaps. It is well known that as a surface plasmon approaches a narrow gap, its group velocity decreases and its electric field increases. Let us treat the plasmons as damped harmonic oscillators linearly coupled and with damping rate γ(*t*) and ω(*t*) as the instantaneous QNM frequency. The temporal evolution of the field is given by 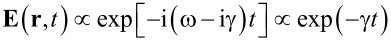, which decays exponentially given γ > 0. Since the plasmon resonances correspond to poles of Green’s function (see Supporting Information File 1), near to such poles, Green’s functions (and consequently, local fields, Equation 6), becomes large. A complex function of such resonances can be found from the position of the corresponding poles in the complex plane frequency ω vs spectral width γ. Assuming γ > 0, we assume a negative sign of the imaginary part of the physical surface frequency. During the field enhancement, the plasmon changes its parameters in space [53], while the QNM frequency is supposed to change monotonically with a decay rate 
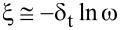
. If both ξ and γ are constants, the time-dependent plasmon amplitude **E**(**r,***t*) is now proportional to e^(ξ−γ)^*^t^* with a competition between the frequency decay rate ξ and the damping rate γ. If ξ−γ > 0, the amplitude of field increases with time. In addition, the plasmon enhancement can be amplified if they are in-phase at a nanogap (hot spots), while the subsequent nanogaps could be out-of-phase, showing a type of dark spot. The spatial distribution of a hot spot surrounded by dark spots is represented in Figure 4.

Another critical factor that affects the localized hot spots is the polarization [13]. Since the plasmon resonance depends on the restoring force resulting from a noncompensated surface charge, this force depends ultimately on the shape of the topography profile and polarization, i.e., it is ruled by the geometrical anisotropy. We can say that the applied polarization of the electric field drives the plasmon resonance to be a geometrical polarization*.* Since the hot spots are strongly dependent on the charge density fluctuations due to rippled surfaces, the effect of the incident light polarization plays a fundamental role. Polarization studies have been performed on plasmonic nanostructures, see, e.g., [54]. While theoretical models have confirmed the experimental observations limited only to perfectly smooth nanogaps with idealized geometry [54–55], other investigations, based essentially on tip-enhanced measurements, take advantage of the so-called across-the-gap polarization, assuming that the strongest enhancement turns up with this polarization direction [54,56].

In our rippled surfaces, light with transverse polarization excites complex plasmon modes with greatly enhanced local fields only for nanogaps that for a local shape reproduce the across-the-gap transverse polarization. Moreover, we found a surprising additional effect: the shape of the gap, but in particular, the depth and width (wall distance) joined to the surrounding gaps play a leading role in the field enhancement. This is clearly a nonlinear effect where the surface geometry is able to confine charge on the walls of a single gap, simultaneously depleting the charge density from the surrounding gaps. Such a nonlinear effect is depicted in Figure 4. We consider a profile extracted from a rippled surface as in Figure 2. Hence, we consider a polarization that is *prevalently* transverse, or longitudinal to the profile. Prevalently means that the polarization, due to irregular ripple spatial configuration, is a mixing of *s*- and *p*-polarized fields, where the *s*- or *p*-direction is referred to the principal axis of the irregular ripples.

To gain insight into the origin of the field enhancement inside the nanogaps, we have computed the local field in the region where the enhancement is the highest, Figure 4. In such nanogaps, the field is mainly concentrated at the middle of the gap. On resonance, surface plasmons are coupled into the gap to excite a localized QNM [57]. In addition, open nanogaps offer also a unique environment to study SPP propagation. SPP propagation is a conceptually different phenomenon from the localized plasmon resonance [58]. However, if the metal–dielectric–metal nanogaps could also represent a sort of waveguide mode for the SPP propagation, the rough surface seems to have a leading role for the formation of the field enhancements in contrast to SPP propagation*.* A unique description of SPP propagation, QNMs in random nanogaps and localized plasmons is highly desirable. This effort will be the natural extension of the results described in this paper.

In turn, the spatial distribution of the nanogaps plays clearly a fundamental role for the formation of hot spots and their relation with subsequent dark modes [59]. A roughness surface configuration with deep ripples is favorable for the presence of many regions with high Γ factors. However, the coupling and the interface with a metallic counterface could reduce the apparent roughness, reducing the field enhancement. This is the case of experimental configurations where an aperture SNOM is used to detect hot spots. The next section is devoted to treat this interesting case.

### Comparison with aperture SNOM experimental measurements

The possibility to correlate topography and optical maps is a key point in assessing localized SPPs and in interpreting the origin of the local optical properties. The capability to correlate topography and optical maps showing the surface electric field is also the principle feature of the SNOM technique. As a consequence, the possibility to perform SNOM measurements on rippled surfaces is a great stimulus to gain insight into how hot spots can be distributed on corrugated surfaces and to locate them relative to the sample morphology.

SNOM can be subdivided into aperture (or apertureless) scattering-SNOM (s-SNOM). Scattering-type SNOM combines the resolution of atomic force microscopy with the sensitivity of optical spectroscopy. In such a configuration, a sharp metal tip is brought within close proximity to a sample surface and illuminated by an external source [60–61]. Following the operating principles as in s-SNOM functionality, tip-enhanced Raman spectroscopy (TERS) involves keeping a metal nanostructure (tip) at a small distance above a sample, providing a highly localized field enhancement. Essentially, in this technique, a single-plasmon-resonant metallic nanostructure is provided in the form of a scanning probe tip of suitable material and geometry. Although TERS has been successfully applied in many applications, resulting in a powerful combination of SERS with Raman–AFM capability, our experimental reference is the aperture-based SNOM technique.

Aperture SNOM in collection mode is, in principle, an excellent tool to identify the sites of local field enhancement (hot spots) and to locate them relative to the sample morphology [62]. There are, however, a few important aspects that must be carefully taken into account when using the SNOM for visualizing hot spots. One of such aspects is the occurrence of rough surfaces, as is typical for rippled samples. When rippled surfaces are investigated, as in our case, the large physical size of the probe apex prevents the topography to be precisely tracked. Since the electric field enhancement due to plasmon resonance depends drastically on the distance from the surface, the probe-to-surface distance is even larger when nanometer-sized grooves, or valleys, are to be analyzed. Ultimately, the spatial resolution of an aperture SNOM measurement is dictated by the size of the tip aperture, which is typically 50 nm diameter. Consequently, strong nanometer-sized hot spots can result in broader and weaker features.

Hence, aperture SNOM requires the average over a surface corresponding to the aperture area, so that Equation 1 is now

[17]
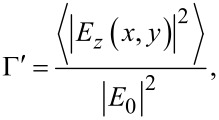


where the brackets denote both the average over the area corresponding to the aperture of the SNOM probe and average time of acquisition for any point, normally on the order of milliseconds. In addition, the *z*-component of the field observed by an aperture SNOM is proportional to *E*_0_(***p***•***n***)(***n***•***e***)exp(−*z*/*d*), where *E*_0_ and ***p*** are the electric field amplitude and the polarization direction for the illuminating light, respectively, *d* is the *z*-depth height of the evanescent electromagnetic field, *z* the SNOM probe–sample distance and ***n*** represents the normal vector to the corrugated surface [38]. The terms ***p***•***n***, and ***n***•***e*** describe the role of the polarization on the efficiency of surface plasmon excitation and the cosine angular distribution of the radiation, respectively [63].

**Figure 5:**
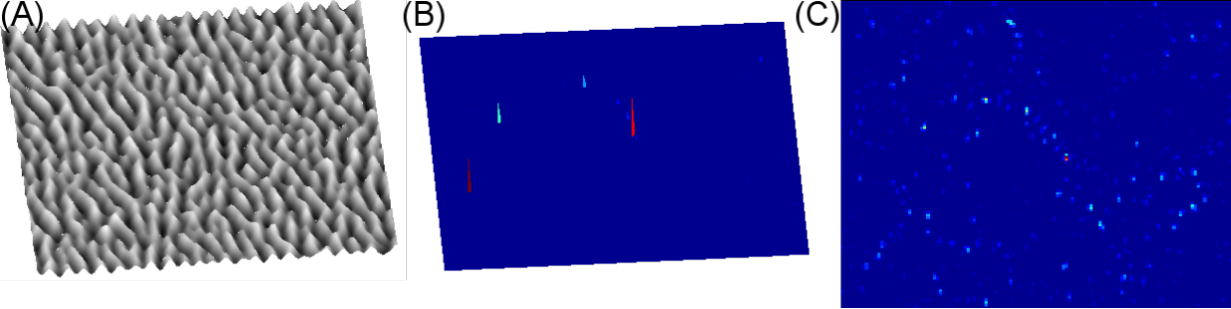
(A) Image of rippled gold surface, 2.5 × 2.5μm and (B) corresponding map of the enhancement factor, maximum Γ ≈ 10^12^, λ = 750 nm. (C) The equivalent map as in (B) but observed by an aperture SNOM measurement (probe of 50 nm diameter) operating in collection mode and positioned at a constant distance of 10 nm over the scanned surface, Γ ≈ 2–5, permittivity of the environment, ε = 1 (air).

The limitations of an aperture SNOM experiment operating in collection mode to investigate hot spots become useful when the system probe plus rippled surface in the condition of the so-called small-roughness limit is analyzed [42]. In Figure 5C the optical map of a rippled surface as recorded by an aperture SNOM measurement is simulated. The measurement simulation provides an aperture SNOM with a probe of 50 nm in diameter, operating in collection mode and positioned at a constant distance of 10 nm over the scanned surface. The external illumination is at λ = 750 nm in order to have a direct comparison with corresponding experimental results [62,64]. The results have been obtained by adopting the small-roughness approximation for the Green’s function.

The results, as shown in Figure 5, display clearly that the aperture SNOM strongly perturbs the hot spot shape and intensity generated when the rippled surface is illuminated by radiation of an appropriate wavelength. The role of the SNOM probe, essentially a metal ring of 50 nm diameter with a dielectric fiber inside, is to reduce the enhancement factor while increasing the number local hot spots, Γ ≈ 2–5, relative to the grooves of the rippled surface. The strong correlation between local hot spots and the rippled pattern evidenced by the SNOM technique can be further usefully employed for the characterization of the optical properties of surface plasmons of random surfaces, given that their tuning is also intrinsically connected to the ripple texture.

## Conclusion

Resonances on surface metallic nanostructures are often found experimentally by probing the structures under investigation with radiation of various frequencies following a trial-and-error method. A general technique for the tuning of these resonances is strongly preferable.

In this paper, we demonstrated the basic connection between the surface-patterned local nanogaps and the general tuning of surface plasmon resonances, both as a function of wavelength and polarization. The effect of nanoscale roughness on such resonances of randomly patterned gold films has been numerically investigated. The field enhancement and its dependence on specific roughness patterns was analyzed producing many different realizations of rippled surfaces.

The ability to reproduce the rippled surfaces led us to opt for a surface integral equations formulation resulting from the application of Green’s theorem on the scattering surfaces directly to the exact electromagnetic wave expression, and hence, the ability to evaluate the corresponding point-by-point enhancement factors. This approach has the advantage that it allows for a much finer spatial discretization, and hence, an improved ability to correlate the hot spots to the surface morphology. The main disadvantage of our method is that since is applied in the frequency domain, we are able to treat only time-harmonic domains, and the solution has to be determined at each single frequency of interest.

We demonstrated that irregular patterns act as metal–dielectric–metal local nanogaps for the resonant plasmonic fields. In turn, the numerical results are compared to experimental data obtained via aperture scanning near-optical microscopy on the spatial localization of the hot spots. Our study could contribute to a better understanding of the role of metallic nanoscale roughness to tune surface plasmon induced hot spots.

## Supporting Information

File 1 Additional Green’s function calculations.
