# Supplementary material for: Near-field surface plasmon field enhancement induced by rippled surfaces
Source: Beilstein J Nanotechnol. 2017 Apr 28;8:956–67. doi: 10.3762/bjnano.8.97 (PMC5433166; doi:10.3762/bjnano.8.97)
Supplement: File 1 — Additional Green’s function calculations. [file Beilstein_J_Nanotechnol-08-956-s001.pdf]

Supporting Information

for

Near-field surface plasmon field enhancement induced by rippled surfaces

Mario D'Acunto*[1,2], Francesco Fuso[3,4], Ruggero Micheletto[5,6], Makoto Naruse[7], Francesco Tantussi[3], and Maria Allegrini[3,4]

Address: [1]CNR-ISM, Istituto di Struttura della Materia, Consiglio Nazionale delle Ricerche, via Fosso del Cavaliere 100, 00133, Rome, Italy, [2]CNR-IBF, Istituto di Biofisica, Consiglio Nazionale delle Ricerche, via Moruzzi 1, 56124, Pisa, Italy, [3]Dipartimento di Fisica Enrico Fermi and CNISM, Università di Pisa, Largo Bruno Pontecorvo 3, 56127, Pisa, Italy, [4]CNR-INO, Istituto Nazionale di Ottica, via Moruzzi 1, 56124, Pisa, Italy, [5]Yokohama City University, Graduate School of Nanobioscience, 22-2 Seto, Kanazawa-ku, Yokohama, Japan, [6]Harvard Medical School, 64 Sydney Street, Suite 170, Cambridge, 02139 MA, USA, and [7]Network System Research Institute, National Institute of Information and Communications Technology, 4-2-1 Nukui-kita, Koganei, Tokyo 184-8795, Japan

Email: Mario D'Acunto* - mario.dacunto@cnr.it

*Corresponding author

Additional Green's Function Calculations

In this section, we calculate the Green's function for the rough surface to be inserted in Eq. (11). Such rough Green's function is given by two main contributions, one is the so-called coherent term and the other one is the fluctuating contribution entirely accounting the roughness surface contribution. The Green's function, G, for a given point source located at $\boldsymbol{r}=\boldsymbol{r}_0$ must satisfy the equation:

$$\left(\nabla^2 + k^2\right)G(\boldsymbol{r},\boldsymbol{r}_0) = -\delta(\boldsymbol{r}-\boldsymbol{r}_0) \qquad \text{(S1)}$$

The rough surface is described by a surface profile z=$h(x,y)$ which is a random function of the surface height. If we consider the value $\boldsymbol{r}_0=\langle h(x,y)\rangle=0$, the boundary conditions provide $G(\boldsymbol{r},\boldsymbol{r}_0)=0$ for $z=\boldsymbol{r}_0$. This constrain can be used to expand the Green's function about the surface height z. Let us consider a one-dimensional case for the surface height, the extension to a bi-dimensional case is immediate, hence the Green's function expanded on z can be written as:

$$G(\boldsymbol{r}_1,\boldsymbol{r}_0) + h(x)\frac{\partial}{\partial z_1}G(\boldsymbol{r}_1,\boldsymbol{r}_0) + \frac{h^2(x)}{2}\frac{\partial^2}{\partial z_1^2}G(\boldsymbol{r}_1,\boldsymbol{r}_0) + \ldots = 0 \qquad \text{(S2)}$$

Keeping the first-order in h in Eq.(S2) and neglecting the higher-order powers of h, and hence making use of the Green's theorem , we approach to the following expression

$$G(\boldsymbol{r},\boldsymbol{r}_0) = G(\boldsymbol{r},\boldsymbol{r}_0=0) + \int_S G(\boldsymbol{r},\boldsymbol{r}_0=0)\left[-\frac{\partial^{\leftarrow}}{\partial z_1}h(x_1)\frac{\partial^{\rightarrow}}{\partial z_1}\right]G(\boldsymbol{r},\boldsymbol{r}_0)d\boldsymbol{s} \qquad \text{(S3)}$$

It should be noted that $G(\boldsymbol{r},\boldsymbol{r}_0=0)$ becomes zero as $\boldsymbol{r}$ approaches to $\boldsymbol{r}_0$, but this is not true inside the integral because the derivative of the same function. The arrows on the derivatives denotes the

direction in which the derivation is operated. At this point, we can calculate the coherent Green's function, that is the evaluation of the Green's function <G($\boldsymbol{r}$,$\boldsymbol{r}_0$)>. Following Ishimaru *et al.*, we apply the Dyson's equation in the condition of the so-called smoothing first-order approximation to obtain [42]:

$$\left\langle G(\boldsymbol{r},\boldsymbol{r}_0)\right\rangle = G(\boldsymbol{r},\boldsymbol{r}_0=0)+\int_S G(\boldsymbol{r},\boldsymbol{r}_2)\left[-\frac{\partial^{\leftarrow}}{\partial z_2}\frac{\partial^2}{\partial z_2 \partial z_1}G(r_2,r_1=0)\left\langle h(x_2)h(x_1)\right\rangle\frac{\partial^{\rightarrow}}{\partial z_1}\right]\left\langle G(\boldsymbol{r}_1,\boldsymbol{r}_0)\right\rangle d\boldsymbol{s}_1 d\boldsymbol{s}_2 \tag{S4}$$

The solution of Eq.(S4) requires the introduction of the spatial Fourier transform described, for the Green's function and spatial correlation, respectively, by $G(\boldsymbol{r},\boldsymbol{r}_0)=\frac{1}{2\pi}\int G(\kappa,z,z_0)e^{i\kappa(z-z_0)}d\kappa$, and $\left\langle h(r_1)h(r_2)\right\rangle=\int\Phi(\kappa)e^{i\kappa(r_1-r_2)}d\kappa$, where $\Phi(\kappa)$ is the spectral density. Note that κ denotes the spatial wavevector. Using the expressions for the Fourier transform and spatial correlation in Eq. (S4), we obtain

$$\left\langle G(\kappa,z,z_0)\right\rangle = G(\kappa,z,z_0=0)+A(\kappa)C(\kappa)\int B(\kappa')\Phi(\kappa-\kappa')d\kappa' \tag{S5}$$

where $A(\kappa)=G(\kappa,z,z_2)\frac{\partial^{\leftarrow}}{\partial z_2}$, $B(\kappa)=\frac{\partial^2}{\partial z_2\partial z_1}G(\kappa,z_2,z_1)$, $C(\kappa)=\frac{\partial^{\rightarrow}}{\partial z_1}\langle G(\kappa,z_1,z_0)\rangle$.

Now, we introduce a reflection coefficient connected to spectral density and plasmon wavevector via the function $\Theta(\kappa)=\tilde{k}\int\tilde{k}\Phi(\kappa-\kappa')d\kappa'$, where $\tilde{k}=\sqrt{k^2-\kappa^2}$,

$$R(\kappa)=\frac{\Theta-1}{\Theta+1} \tag{S6}$$

It should be noted that for Θ(*κ*)=−1, the reflection coefficient has a pole. For this pole the rough surface gives a relevant contribution to field enhancement. Finally, adopting the spatial Fourier transform representation for G_0 and coherent the Green's functions written as

$$G_0(\kappa;z,z_0)=\frac{i}{2k_z}\left[e^{ik_z|z-z_0|}-e^{ik_z|z+z_0|}\right] \qquad \langle G(\kappa;z,z_0)\rangle=\frac{i}{2k_z}\left[e^{ik_z|z-z_0|}+R(\kappa)e^{ik_z|z+z_0|}\right] \quad \text{(S7)},$$

and taking in mind that $\frac{1}{2\pi}\int\frac{i}{2k_z(\kappa)}\left(e^{ik_z|z-z_0|}+R(\kappa)e^{ik_z|z+z_0|}\right)d\kappa=\frac{i}{4}H_0^1(kR(\kappa))$, where H_0^1 is the Hankel function of first kind and order zero, after some simple algebraic calculations and invoking residue evaluation of reflection coefficient poles, we achieve to Eq. (9) for the coherent case and Eq. (12) for the fluctuating one.
